# Simultaneous resection of colorectal cancer and liver metastases

**DOI:** 10.1590/0102-67202025000014e1883

**Published:** 2025-07-21

**Authors:** Raphael Leonardo Cunha ARAUJO, Marcelo Moura LINHARES, Antonio Roberto Franchi TEIXEIRA, Aleksandar KARAMARKOVIC, Hugo Pinto MARQUES, Timothy M. PAWLIK, Rene ADAM, Olivier SOUBRANE, Maria Ignez BRAGHIROLI, Paulo HERMAN, Ricardo Lemos COTTA-PEREIRA, International Hepato-Pancreato-Biliary Association

**Affiliations:** 1 Universidade Federal de São Paulo, Department of Surgery – São Paulo (SP), Brazil.; 2 Instituto do Fígado – Campinas (SP), Brazil.; 3 University of Belgrade, Medical Faculty – Belgrade, Serbia.; 4 Centro Hospitalar Universitário de Lisboa Central, Curry Cabral Hospital, Hepato-Biliary-Pancreatic and Transplantation Centre – Lisbon, Portugal.; 5 Ohio State University, Wexner Medical Center, Department of Surgery – Columbus (OH), USA.; 6 University Paris-Saclay, AP-HP Paul Brousse Hospital, Hepato Biliary Surgery, Cancer and Transplantation Unit – Villejuif, France.; 7 Universite Paris Descartes, Institute Mutualiste Montsouris, Oncologic and Metabolic Surgery, Department of Digestive – Paris, France.; 8 Universidade de São Paulo, Institute of Cancer of São Paulo – São Paulo (SP), Brazil;; 9 Universidade de São Paulo, Faculty of Medicine, Department of Gastroenterology – São Paulo (SP), Brazil.; 10 D’Or Institute for Research and Education, Digestive Surgery Residency Program – Rio de Janeiro (RJ), Brazil.

**Keywords:** Neoplasm Metastasis, Liver, General Surgery, Hepatectomy, Colectomy, Metástase Neoplásica, Fígado, Cirurgia Geral, Hepatectomia, Colectomia

## Abstract

Although the presence of synchronous colorectal liver metastases (CRLM) represents an important prognostic factor for recurrence-free survival (RFS) and overall survival (OS), the definitions of synchronicity are variable in the literature, including metastases at the time of diagnosis, or even before the diagnostic of the primary site of colorectal cancer (CRC), until either six or 12 months after the time of diagnosis, according to the author of each study. Simultaneous approaches to treat CRC and CRLM seem to be safe for patients carefully selected without jeopardizing oncologic outcomes, with similar complication rates, shorter hospital length of stay, and operation times even for major hepatectomies. However, there is no consensus about the optimal timing to approach the primary tumor and CRLM, whether simultaneously or staged, and both performance status and the presence of symptoms play important roles in the treatment sequence, perhaps avoiding two high-risk procedures at the same time.

## HIGHLIGHTS

- Synchronous colorectal liver metastases represents an important prognostic factor for recurrence-free survival and overall survival
- Simultaneous approaches to treat colorectal cancer and colorectal liver metastases seem to be safe for patients carefully selected
- There is no consensus about the optimal timing to approach the primary tumor and colorectal liver metastases

## CENTRAL MESSAGE

The presence of synchronous colorectal liver metastases represents an important prognostic factor for recurrence-free survival and overall survival, the definitions of synchronicity are variable in the literature, including metastases at the time of diagnosis, or even before the diagnostic of the primary site of colorectal cancer, and until either six or 12 months after the time of diagnosis, according to the authors of the studies.

## PERSPECTIVES

Simultaneous approaches to treat colorectal cancer and colorectal liver metastases seem to be safe for patients carefully selected without jeopardizing oncologic outcomes, with similar complication rates, shorter length of stay and operation times even for major hepatectomies. However, there is no consensus about the optimal timing to approach the primary tumor and colorectal liver metastases, whether simultaneously or staged, and both performance status and presence of symptoms play important roles in the treatment sequence, perhaps avoiding two high-risk procedures at the same time

## VISUAL ABSTRACT

**Figure:**
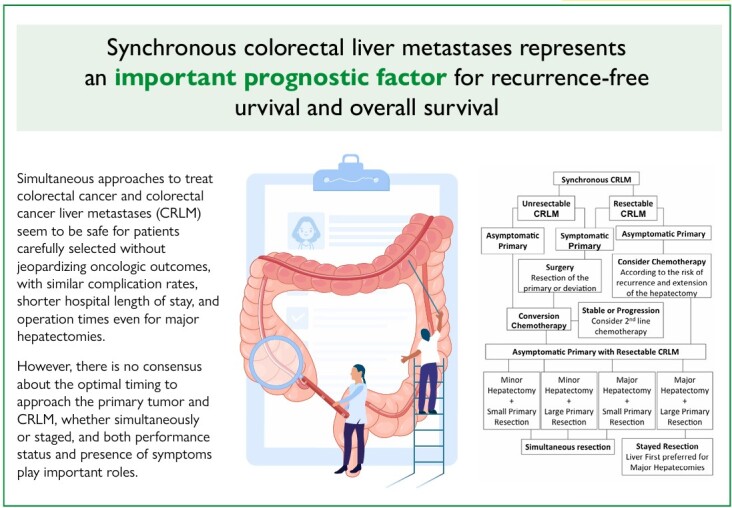

## RESUMO

Embora a presença de metástases hepáticas colorretais síncronas (MHCS) represente um fator prognóstico importante para a sobrevida livre de recorrência (SLR) e sobrevida global (SG), as definições de sincronicidade são variáveis na literatura, incluindo metástases no momento do diagnóstico, ou mesmo antes do diagnóstico do sítio primário do câncer colorretal (CCR), e até seis ou doze meses após o momento do diagnóstico, de acordo com as designações do autor de cada estudo. Abordagens simultâneas para tratar CCR e MHCS parecem ser seguras para pacientes cuidadosamente selecionados sem comprometer os resultados oncológicos, com taxas de complicações semelhantes, menor tempo de internação hospitalar e tempos de operação, mesmo para hepatectomias maiores. No entanto, não há consenso sobre o momento ideal para abordar o tumor primário e o MHCS, seja simultaneamente ou em estágios, e tanto o status de desempenho quanto a presença de sintomas desempenham papéis importantes na sequência do tratamento e talvez evitando dois procedimentos de alto risco ao mesmo tempo.

## INTRODUCTION

 Although the presence of synchronous colorectal liver metastases (CRLM) represents an important prognostic factor for recurrence-free survival (RFS) and overall survival (OS), the definitions of synchronicity are variable in the literature, including metastases at the time of diagnosis, or even before the diagnostic of the primary site of colorectal cancer (CRC), and until either six or 12 months after the time of diagnosis, according to the authors of the studies^1,11^. This review highlights and discusses oncological and technical aspects of the decisionmaking process for indications of simultaneous resection of CRLM and CRC. 

### The standard multimodal approach

 Patient selection for systemic or surgical treatment of CRLM has been made based on image workouts, laboratory tests, clinical presentation, and performance status of the patients. Fong et al. described a clinical risk score (CRS) for the recurrence of CRLM which includes the presence of synchronous disease as an independent prognostic factor, that predicts the risk of recurrence after curative-intent hepatic resection of CRLM in 1,001 patients^11^ . The score count is one point for each criterion: presence of positive nodal status of the primary tumor, disease-free interval (DFI–from primary to CRLM) until 12 months, number of hepatic lesions >1, preoperative carcinoembryonic antigen (CEA) level >200 ng/mL, and size of the largest tumor >5 cm with a score ranging from 0 to 5, with OS rates ranging from 74 to 14 months respectively^11^ . For the purposes of this review, patients with scores of 0, 1, or 2 were deemed to have a low risk of recurrence, whereas patients with scores of 3, 4, or 5 were deemed to have a high risk of recurrence. 

### The timing of surgery

 CRC and CRLM appear to have no effect on oncologic outcomes. Brouquet et al. reported a series of 142 patients with synchronous CRLM who underwent three different curative-intent approaches: 72 patients underwent the classic approach (CRC first), 43 underwent a combined strategy (both CRC and CRLM), and 27 received a reverse approach (CRLM first)^10^ . No differences in surgical and oncologic outcomes were observed. Nevertheless, patient selection bias was based on the median number of CRLM per patient: one in the combined group, three in the classic group, and four in the reverse strategy group (p=0.01 classic vs. reverse; p<0.001 reverse vs. combined) were detected. Moreover, Silberhumer et al. published two studies showing neither harm nor benefit for both surgical and oncologic outcomes for patients undergoing simultaneous resection of rectal cancer and CRLM^16,17^. The authors reported simultaneous procedures performed by a midline incision and including minor and major hepatectomies (three or more segments), and the surgical treatment of rectal lesions mainly consisted of low anterior resection (90% for simultaneous versus 76% for staged; p<0.01)^17^ . Considering only patients who underwent major hepatectomies, the simultaneous procedures group presented shorter operative time (6.9±1.3 vs. 9.2±2.3 days, p<0.01), and shorter hospital length of stay (LOS) (12±5 vs. 18±7 days; p<0.01)^17^ . More recently, in a randomized clinical trial (RCT), Boudjema et al. revealed that complication rates did not appear to differ when colorectal cancer and synchronous liver metastases are resected simultaneously; however, the simultaneous approach provided gains in long-term outcomes^9^ . Indeed, it seems that the CRLM burden and presence of symptoms for CRC play a significant role in the choice of either reverse, combined, or classic strategies, and all of them are possible to promote a curative-intent treatment^7,10 ,19^. The presence of symptoms (generally bleeding or obstruction) is one of the most important factors in the management of these patients, and it works similarly for both rectal and colon cancer, in terms of the need for deviation, colonic stent, or resection upfront of the primary site. Postponing the definitive treatment of the CRC and the CRLM in symptomatic patients may be done to grant a final goal of curative-intent treatment including both surgical sites and systemic treatment. 

### The systemic treatment

 It is part of the curative-intent treatment of CRLM and putatively helpful as a patient-select tool in asymptomatic patients, and its upfront use has been advocated for patients with high risks of recurrence^5,7,15,18^. The question of whether chemotherapy should be delivered either before and after or only after liver surgery remains an open debate, and there is no RCT on this issue^12^ . A series of 411 patients with initially resectable CRLM approaching patient selection and chemotherapy regimen in ten years of practice at Memorial Sloan Kettering Cancer Center demonstrated no differences in OS. Nevertheless, the RFS was significantly better for those in the adjuvant chemotherapy group than for the perioperative regimen group (5-year RFS of 38 and 31%, respectively, p=0.036, p<0.05)^3^ . Unsurprisingly, once the RFS was adjusted by low and high CRS, the differences between groups vanished, and the difference between groups was no longer significant. Concisely, patients at high risk of recurrence, postoperative liver failure due to borderline small remnant liver volume, and higher tumor burden should not be operated on upfront because both tumor biology and chemo response can be tested; consequently, fine-tuning patient selection eventually results in CRLM downsizing for "good responders," or occasionally forgoes an unnecessary surgery in "bad responders". 

### Minimally-invasive surgery (MIS)

**Figure 1:**
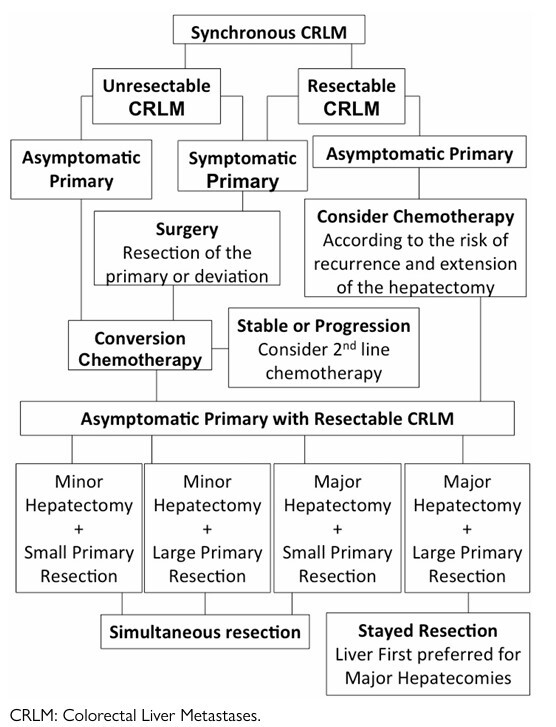
This flowchart represents the decision-making process for the simultaneous surgical treatment of resectable colorectal liver metastases based on clinical presentation and its risk of complications and recurrence^6^.

 Considering the laparoscopic approach, simultaneous resections have also been attempted with success in case reports and small series; they seem to be more conservative regarding the extension of the liver resection^4^. We previously suggested a flowchart based on a review of the sparse literature addressing the simultaneous laparoscopic approach as an attempt to aid the decision-making process based on the initial clinical presentation of synchronous CRLM, as shown in Figure 1 ^4^. Nevertheless, for both open and minimally-invasive approaches, the liver-first approach should be considered if CRLM represents extensive resection, avoiding occasional progression to unresectable lesions, perhaps small lesions with unfavorable anatomical relations. 

 Considering the sequencing of the simultaneous procedure, liver resection first facing the relative hypotension during the induced low central venous pressure to avoid bleeding from hepatic veins can be minimized with fluid resuscitation during the rectal portion of the procedure, reducing the risk of transient pre-renal failure^14^. Although the Pringle maneuver has no effect on long-term outcomes, it appears to reduce intra-operative bleeding caused by portal vein and the hepatic artery occlusion; however, it may increase the risk of acidosis caused by liver reperfusion^21^. Additionally, if the rectal was performed before the liver resection, the venous congestion from the Pringle maneuver could jeopardize a recently created bowel anastomosis^17^. 

### The imaging resources

 Computerized tomography (CT), magnetic resonance imaging (MRI), positron emission tomography (PET) scan and intraoperative ultrasound (IOUS) are the imaging resources to evaluate CRLM. Regarding the preoperative use of chemotherapy, radiographic partial or complete responses, with tumor shrinkage or disappearance of CRLM, do not necessarily mean a pathologic response^6^. With pooled sensitivity estimates of 85.7, 69.9, and 54.5%, respectively, the use of MRI appears to be the most appropriate modality for assessing CRLM^20^. Furthermore, using a liver-specific MRI contrast agent (gadoxate disodium) and diffusion-weighted MRI, MRI has the potential to improve detection rates in a subset of patients who have undergone chemotherapy, with the accuracy of 89.2, 76.5, and 65.1%, respectively, among contrast-enhanced (CE) plus diffusion-weighted MRI, CE MRI, and diffusion-weighted MRI^13^. While PET scanning has a high sensitivity to detect CRC, it does not supplant a CE CT or MRI to localize small lesions and plan resection; thus, it seems better indicated to evaluate possible extrahepatic disease, and the previous use of chemotherapy must be considered as a flaw in the detection of the small lesions contrasting with the high background of glucose uptake in hepatocytes, consequently limiting the detection of metastases smaller than 1 cm in size^2^. Nevertheless, the use of CE MRI does not exclude the necessity of both IOUS and CE-IOUS for intraoperative detection and surgical planning of CRLM resection, with the accuracy of CE-IOUS higher than IOUS, CE-MRI, and CE-CT being 97, 88, 83, and 81%, respectively^8^. Moreover, the IOUS can be optimized using image fusion systems, which promote IOUS navigation by CT or MRI, creating and course-plotting a map of areas of interest that can help to identify missing metastases that were not seen on preoperative imaging of patients who had undergone preoperative chemotherapy^6^. Other strategies to avoid disappearing CRLM, and occasionally to evaluate progression during chemotherapy, are to use a short interval for imaging surveillance (2–3 months), and to use a marking technique for intraoperative localization of small lesions before systemic chemotherapy, in which coils are placed behind the deep margin of the small lesions at risk of disappearing^6,7,22^. 

### Simultaneous approaches

 Thus, simultaneous approaches to treat CRC and CRLM seem to be safe for patients carefully selected without jeopardizing oncologic outcomes, with similar complication rates, shorter LOS and operation times even for major hepatectomies. However, there is no consensus about the optimal timing to approach the primary tumor and CRLM, whether simultaneously or staged, and both performance status and presence of symptoms play important roles in the treatment sequence, perhaps avoiding two high-risk procedures at the same time. 

## AUTHORS’ COMMENTS

 Rene Adam (upfront resection of primary tumor and metastasis): There is no place for this approach because we do not know anything about this disease, which is located in at least two tumor sites. It is necessary to give neoadjuvant chemotherapy before knowing what to do. There is a sort of “fire in the liver and fire in the colon or in the rectum”. We should stop the fire in the two sites to do the surgery in good conditions. 

 Timothy M. Pawlik: For patients very symptomatic with synchronous disease, obviously there is an indication to deal with the primary beforehand. And, typically, we would do a colonoscopy with pediatric colonoscope. If the endoscopist can get a pediatric colonoscope by it, then we are going to be okay. Because usually chemotherapy is so effective that not only will there be a response in liver but usually the primary also responds. But if they cannot get a pediatric colonoscope through it, then typically we would take out the primary. And/or if it was amenable to a stent but something probably would need to be done because our medical oncologist would be nervous about initiating chemotherapy and then the patient obstructing during chemotherapy, and then you are in a whole another situation. 

 Hugo Pinto Marques: Usually we choose neoadjuvant chemotherapy in patients that have a more aggressive biology. And synchronous presentation usually is like that. So, conceptually, synchronous presentation should be at a point an indication for neoadjuvant chemotherapy. We have to take into account several other factors such as the size and number of the metastases, the expression of tumor markers, etc. So, I do not think there is a clear-cut answer. For some low-burden diseases that have no signs of aggressiveness, we can consider upfront resection for synchronous metastasis. 

 Rene Adam (simultaneous resection—open or minimally-invasive surgery): There are so many questions in synchronous metastasis. First, should we remove the two sites at the same time? Should we do it by minimally-invasive surgery? Of course, this depends for me. The ideal laparoscopic indication is when you have relatively limited hepatic disease, and you know in advance, very clearly, what you should do. When you have any doubt about the type of surgery you should do, much better open. 

 Paulo Herman (magnetic resonance imaging and Primovist): We do it routinely in multiple nodules. In a single nodule, the classic magnetic resonance imaging is okay. 

 Hugo Pinto Marques: I do think that intraoperative ultrasound is mandatory for all cases. There is a limitation of intraoperative ultrasound when you use a minimally-invasive surgery. However, I think there are some advantages with this approach and so we can minimize this lack of efficacy of the laparoscopic intraoperative ultrasound with excellent imaging. We try to have MRI with Primovist in patients that are submitted to MIS. I think the studies comparing open with minimally-invasive surgery for liver metastasis are essentially for low-burden disease. And, in fact, it is at least not inferior but I’m not so sure that there is an advantage in cases with a higher burden of disease. I would say that the limitations of intraoperative ultrasound and the higher number of metastases might render the MIS, in this setting, inferior. So, I would favor an open approach in the presence of five or more metastases, usually. 

 Rene Adam (liver or colon first?): I think it depends on the extent of both sites. Usually, we have multiple metastases in the liver and an asymptomatic primary lesion. At that time, it seems logical and it has been proved by a recent paper by Felice Giuliante on LiverMetSurvey that the best approach for bilateral multiple metastases is to begin by the liver. Now, if you have only one or two lesions in the liver and a complex rectal surgery, it could make sense to begin by the rectum to be sure that you do a curative resection of the primary and so on. 

 Maria Ignez Braghiroli (chemotherapy): So, it depends on the volume of disease. If this is a patient that already has a resectable disease and we do not need much response, which is when I get to know the biology of that disease, we would probably just do leucovorin, 5-fluorouracil and oxaliplatin (FOLFOX) and we’ll re-stage in either three or four cycles, which means more or less two months of treatment. We do not want to overtreat these patients with oxaliplatin before they go to surgery. If we think we need some response, or it’s a right colon and depending on the molecular biology, if it is RAS proteins (proto-oncogenes), rapidly accelerated linfosarcoma (RAF) wild type or mutated ("an essencial serine/threonine kinase constituent of the mitogen-activated protein kinase pathway and a downstream pathway and a downstream effector of the central signal transduction mediator RAS"), then we would decide on a more intense treatment, maybe leucovorin, 5-fluorouracil, irinotecan hydrochloride and oxaliplatin (FOLFIRINOX), and then restage again in more or less two months. However, if we think these nodules are tiny and they might disappear on imaging in around two months of treatment, then we decide if we should go straight to surgery or if we should do some more treatment. 

 It is controversial if we should do an adjuvant treatment. There is a Japanese trial suggesting that we should not continue with chemotherapy after the resection, but since there is high risk of recurrence and it depends on the patient’s tolerance to the treatment, most of us would probably offer some complementation of chemotherapy for a few months. Not more than six, for sure. 

 Timothy M. Pawlik: There is a study by MD Anderson talking about the use of liquid biopsy (circulating tumor DNA—ctDNA) to inform post-operative treatment. So, in this trial, patients are getting upfront chemotherapy and then they get surgery and those who are then ctDNA negative are going, kind of going on chemo with Xeloda. But if you are ctDNA positive, then you are going to get the traditional chemotherapy. In the future, maybe ctDNA will help better inform who truly needs additional chemotherapy in the post-operative setting. 

## Data Availability

The datasets generated and/or analyzed during the current study are available from the corresponding author upon reasonable request.

## References

[1] Adam R, Gramont A, Figueras J, Kokudo N, Kunstlinger F, Loyer E (2015). Managing synchronous liver metastases from colorectal cancer: a multidisciplinary international consensus. Cancer Treat Rev.

[2] Akhurst T, Kates TJ, Mazumdar M, Yeung H, Riedel ER, Burt BM (2005). Recent chemotherapy reduces the sensitivity of [18F]fluorodeoxyglucose positron emission tomography in the detection of colorectal metastases. J Clin Oncol.

[3] Araujo R, Gönen M, Allen P, Blumgart L, DeMatteo R, Fong Y (2013). Comparison between perioperative and post-operative chemotherapy after potentially curative hepatic resection for metastatic colorectal cancer. Ann Surg Oncol.

[4] Araujo RL, Gönen M, Herman P (2015). Chemotherapy for patients with colorectal liver metastases who underwent curative resection improves long-term outcomes: systematic review and meta-analysis. Ann Surg Oncol.

[5] Araujo RL, Riechelmann RP, Fong Y (2017). Patient selection for the surgical treatment of resectable colorectal liver metastases. J Surg Oncol.

[6] Araujo RLC, Figueiredo MN, Sanctis MA, Romagnolo LGC, Linhares MM, Melani AGF (2020). Decision making process in simultaneous laparoscopic resection of colorectal cancer and liver metastases. Review of literature. Acta Cir Bras.

[7] Araujo RLC, Milani JM, Armentano DP, Moreira RB, Pinto GSF, Castro LA (2020). Disappearing colorectal liver metastases: Strategies for the management of patients achieving a radiographic complete response after systemic chemotherapy. J Surg Oncol.

[8] Arita J, Ono Y, Takahashi M, Inoue Y, Takahashi Y, Matsueda K (2015). Routine preoperative liver-specific magnetic resonance imaging does not exclude the necessity of contrastenhanced intraoperative ultrasound in hepatic resection for colorectal liver metastasis. Ann Surg.

[9] Boudjema K, Locher C, Sabbagh C, Ortega-Deballon P, Heyd B, Bachellier P (2021). Simultaneous versus delayed resection for initially resectable synchronous colorectal cancer liver metastases: a prospective, open-label, randomized, controlled trial. Ann Surg.

[10] Brouquet A, Mortenson MM, Vauthey JN, Rodriguez-Bigas MA, Overman MJ, Chang GJ (2010). Surgical strategies for synchronous colorectal liver metastases in 156 consecutive patients: classic, combined or reverse strategy?. J Am Coll Surg.

[11] Fong Y, Fortner J, Sun RL, Brennan MF, Blumgart LH (1999). Clinical score for predicting recurrence after hepatic resection for metastatic colorectal cancer: analysis of 1001 consecutive cases. Ann Surg.

[12] Khessairi N, Mallek I, Mbarek M, Zaafouri EB, Gharbi L, Boufaroua AL (2024). Neoadjuvant treatment of liver metastases of colorectal cancer: predictive factors of pathological response. Arq Bras Cir Dig.

[13] Macera A, Lario C, Petracchini M, Gallo T, Regge D, Floriani I (2013). Staging of colorectal liver metastases after preoperative chemotherapy. Diffusion-weighted imaging in combination with Gd-EOB-DTPA MRI sequences increases sensitivity and diagnostic accuracy. Eur Radiol.

[14] Melendez JA, Arslan V, Fischer ME, Wuest D, Jarnagin WR, Fong Y (1998). Perioperative outcomes of major hepatic resections under low central venous pressure anesthesia: blood loss, blood transfusion, and the risk of postoperative renal dysfunction. J Am Coll Surg.

[15] Nordlinger B, Sorbye H, Glimelius B, Poston GJ, Schlag PM, Rougier P (2013). Perioperative FOLFOX4 chemotherapy and surgery versus surgery alone for resectable liver metastases from colorectal cancer (EORTC 40983): long-term results of a randomised, controlled, phase 3 trial. Lancet Oncol.

[16] Silberhumer GR, Paty PB, Denton B, Guillem J, Gonen M, Araujo RLC (2016). Long-term oncologic outcomes for simultaneous resection of synchronous metastatic liver and primary colorectal cancer. Surgery.

[17] Silberhumer GR, Paty PB, Temple LK, Araujo RL, Denton B, Gonen M (2015). Simultaneous resection for rectal cancer with synchronous liver metastasis is a safe procedure. Am J Surg.

[18] Sonbol MB, Siddiqi R, Uson PLS, Pathak S, Firwana B, Botrus G (2022). The role of systemic therapy in resectable colorectal liver metastases: systematic review and network meta-analysis. Oncologist.

[19] Torres OJM, Torzilli G, Enne M, Gonçalves R, Santibanes E, Pawlik T (2025). Surgical techniques to increase resect ability in liver metastasis. Arq Bras Cir Dig.

[20] van Kessel CS, Buckens CFM, van den Bosch MAAJ, van Leeuwen MS, van Hillegersberg R, Verkooijen HM (2012). Preoperative imaging of colorectal liver metastases after neoadjuvant chemotherapy: a meta-analysis. Ann Surg Oncol.

[21] Weiss MJ, Ito H, Araujo RLC, Zabor EC, Gonen M, D’Angelica MI (2013). Hepatic pedicle clamping during hepatic resection for colorectal liver metastases: no impact on survival or hepatic recurrence. Ann Surg Oncol.

[22] Zalinski S, Abdalla EK, Mahvash A, Vauthey JN (2009). A marking technique for intraoperative localization of small liver metastases before systemic chemotherapy. Ann Surg Oncol.

